# Accessing Neuromarketing Scientific Performance: Research Gaps and Emerging Topics

**DOI:** 10.3390/bs12020055

**Published:** 2022-02-21

**Authors:** Lucília Cardoso, Meng-Mei Chen, Arthur Araújo, Giovana Goretti Feijó de Almeida, Francisco Dias, Luiz Moutinho

**Affiliations:** 1 CiTUR-Leiria, 2411-901 Leiria, Portugal; lucilia.a.cardoso@ipleiria.pt (L.C.); francisco.dias@ipleiria.pt (F.D.); 2 Ecole Hoteliere de Lausanne, HES-SO University of Applied Sciences and Arts Western, 2800 Delémont, Switzerland; meng-mei.chen@ehl.ch; 3 Transdisciplinary Research Center on Innovation & Entrepreneurship Ecosystems (TRIE), Lusofona University, 1749-024 Lisboa, Portugal; p5706@mso365.ulp.pt; 4 Visiting Professor of Marketing, Suffolk Business School, University of Suffolk, Suffolk IP4 1QJ, UK; luizammoutinho@gmail.com

**Keywords:** neuromarketing scientific performance, research gaps, emerging topics, SciVal topic prominence, bibliometric analysis

## Abstract

(1) Background: Using neuroscience to understand and influence consumer behavior often leads to ethical controversy. Thus, it is necessary to demystify the use of neuroscience for marketing purposes; the present paper, by accessing the worldwide academic performance in this domain, fulfills this objective. (2) Methods: All extant literature on neuromarketing indexed to the Scopus database—318 articles—was subjected to a bibliometric analysis through a mixed-method approach. (3) Results: The results show that Spain leads the ranks of the most productive countries, while Italian researchers clearly dominate in terms of collaboration. Regarding the most prominent topics, the connection between “Neuroscience” and “Advertising” is highlighted. The findings provide a better understanding of the state-of-the-art in neuromarketing studies, research gaps, and emerging research topics, and additionally provide a new methodological contribution by including SciVal topic prominence in the bibliometric analysis. (4) Conclusions: As practical implications, this study provides useful insights for neuromarketing researchers seeking funding opportunities, which are normally associated with topics within the top prominence percentile or emerging topics. In terms of originality, this study is the first to apply SciVal topic prominence to a bibliometric analysis of neuromarketing, and provides a new bibliometric indicator for neuromarketing research.

## 1. Introduction

As a research field, neuromarketing emerged from the application of neuroscience methods and techniques for marketing purposes, i.e., consumer behavior as a response to a certain stimulus [1]. In this context, neuromarketing is an interdisciplinary domain with great of potential, as it allows researchers to understand and predict consumer choices and behavior. Therefore, it can lead to new marketing theories and good practices. However, the fulfillment of such potential is often limited by a mystified view of the use of neuroscience methods by marketing researchers. In order to mitigate this problem, Ref. [2] mapped the extant literature on neuromarketing, documenting the different methods employed and identifying the main researchers on the topic.

Mapping scientific literature through bibliometric studies is a useful method of providing an assessment of scientific production in a specific area over a given period [3]. Bibliometric studies allow for the assessment of research, including the themes sought, methods employed, and samples used. In this context, Koseoglu [4] argues that bibliometric studies highlight unknown patterns in research fields or disciplines, helping researchers to develop theories and test hypotheses, concluding that “these structural, dynamic, evaluative, and predictive models can be skillfully applied in the evaluation and prediction of a field” (p. 191).

Aware of this potential, several other studies have mapped scientific production on neuromarketing. Amongst these, Alsharif et al. [5] analysed studies indexed to the Web of Science database published between 2007 and 2019. In addition to being limited to this database and time period, the study did not include a key-theme analysis. Sousa and Lara [6], in turn, conducted a bibliometric study to identify the technologies applied in neuromarketing. This study, however, did not include articles indexed in the Scopus database. Finally, Wannyn [7] analysed studies from Web of Science covering the 2004–2015 period. In sum, to the best of the authors’ knowledge no previous work has mapped neuromarketing research production in the Scopus database. Moreover, no studies dealing with the mapping of co-word analysis and SCival topic prominence in neuromarketing were found. SCival topic prominence is provided by Scopus and combines three metrics that indicate the momentum of a topic: (1) Citation count in year n of papers published in n and n − 1; (2) Scopus view count in year n to papers published in n and n − 1; and (3) Average CiteScore for year n [8,9,10].

To contribute to the filling of these research gaps, the present study aims to assess the global performance of neuromarketing studies. To achieve this general goal, the following specific objectives were adopted:

(1) To determine the overall performance of neuromarketing research;
(2) To determine the performance of the most cited articles;
(3) To identify the most prominent topics within the neuromarketing field;
(4) To identify the prominent Scival topics on neuromarketing; and
(5) To identify the best Scival topics on neuromarketing by percentile.

In order to fulfill these specific objectives, all of the extant literature on neuromarketing indexed to the Scopus database was analyzed. Said body of literature included a total of 318 articles published during the 2007–2020 period. The retrieved articles were subjected to a bibliometric analysis through a mixed-method approach, which encompassed a key-word analysis and a SCival topic prominence analysis. The findings revealed the overall structure of the neuromarketing topic as well as research gaps and emerging research areas, which is useful for both researchers and research institutions. Moreover, the authors with highest research performance were identified, and collaboration patterns were revealed.

Regarding the topic’s overall performance, the findings point to the most productive journals, countries, research institutions, and researchers, and provide a general picture of global collaboration. In terms of the most cited articles, the findings highlight the top ten papers in terms of views and citation, amongst which, as in other emergent topics, introductory works and systematic reviews are dominant. In terms of the most prominent topics, the findings point to the connection between “Neuroscience”, “Advertising”, “Television”, and several topics related to neuroscience techniques. The relationship between these topics is addressed in more detail in the conclusions. Finally, regarding the top 1% percentile, the findings show that these studies consist of audience response studies, methodology enhancement studies, and literature reviews. These findings provide a general picture of the most promising topics in neuromarketing, which is particularly useful for individual researchers and research centers aiming to increase their own impact factor and maximize their chances of obtaining funding for their projects.

## 2. Mapping Neuromarketing Science

### 2.1. Bibliometric Studies on Neuromarketing

Neuroscience methods use tools and techniques to measure, map and record brain or neural activity. In doing so, they generate neurological representations of this activity, which allows scientists to understand specific responses in the brain and nervous system upon exposure to a certain stimulus [1]. Due to their effectiveness in explaining human behavior, these methods are employed in several research fields. Recently, they have begun to be applied in order to “analyze and understand human behavior related to markets and marketing exchange” [11] (p. 3803), originating a new research field: neuromarketing [12]. In this context, neuromarketing is an interdisciplinary domain in which theories and methods from neuroscience and marketing, as well as from related disciplines such as economics, psychology and tourism, are combined in order to understand consumer choices and behavior. Neuromarketing is thus a promising field, as findings in the area can lead to new foundations and generate new marketing theories or complement existing ones [2]. The majority of neuromarketing studies focus on emotions, which play an important role in all human activities. Emotions relate to various affective states, both positive and negative [13], as well as to the psycho-behavioral aspects of consumers.

As argued by Lim [2], “neuromarketing, as a method of investigation, is important because it uses neuroscientific theories and methods to gain access to otherwise hidden information” (p. 1). However, the same author argues that neuromarketing needs to be demystified. Although there are many conceptual works and several bibliographical reviews on the topic, few studies have rigorously produced empirical findings. Therefore, Lim [2] points out a lack of effectiveness in the use of neuroscience measurement techniques to advance marketing theory and address ethical issues. Moreover, neuroscience can intimidate academics due to lack of knowledge regarding the data-gathering methods it uses. In this context, Lim [2] attempts to enhance understanding of the potential of neuromarketing by documenting the different neuroscientific methods used in neuromarketing, identifying the main researchers on the topic, and addressing their main contributions. In other words, the study mapped the extant literature on neuromarketing through a bibliometric study, the same method employed in the present study. Therefore, the next section is dedicated to addressing the main fundaments and contributions of the application of bibliometric methods to the mapping of research topics.

### 2.2. Mapping Research Topics through Bibliometric Studies

Bibliometric studies can analyze several aspects of a body of research, including the productivity of individual scholars and institutions, knowledge flow and social networks, topics’ long-term development trends and emerging topics, journal rankings and journal development, and the most frequently cited scholars and works [14]. Koseoglu et al. [4] and Huang et al. [15] identified three types of bibliometric methods: relational techniques, evaluative techniques, and review studies. Relational techniques explore relationships within research, such as the structure of research fields, the emergence of new research themes and methods, and co-citation and co-authorship patterns. These can be divided into four categories: co-citation analysis, co-word analysis, co-authorship analysis, and bibliographic coupling [4] (p. 182). Evaluative techniques assess the impact of scholarly work, usually aiming to compare the performance or scientific contributions of two or more individuals or groups. To this end, evaluative techniques employ productivity measures, impact metrics, and hybrid metrics. Finally, review studies simply perform thorough literature reviews on a given topic. Through these three sets of techniques, bibliometric studies allow researchers to analyze research topics and the publications of individuals and/or institutions, as well as to map the structure and dynamics of science [15]. In sum, they serve to assess research performance.

Research performance is characterized by metrics that measure certain variables considered to define academic excellence. The term is associated with many programs and departments of institutions that are recognized as having high-quality research output [16]. Quantitatively, research performance is assessed by a range of metrics such as citations per article [17] and other citation metrics [18], cooperation indicators [19], and institutional contribution [20]. Additionally, it can be assessed through qualitative performance aspects [21].

When applied to a country, research performance can be defined by the performance of its institutions, which in turn depends on the performance of its researchers and is frequently conveyed through rankings (i.e., authors, affiliations, journal scores, number of articles published) [22,23]. More recently, research productivity has been closely associated with collaboration between authors and institutions [23], which has been shown to be a tool for knowledge creation, acquisition, and dissemination [24]. In this context, techniques focused on cooperation, such as network analysis, have been incorporated into bibliometric studies.

Adopting a broader approach to bibliographic analysis, the resent studies of Cardoso et al. [8,9,10], and Lima Santos et al. [25] have added a qualitative aspect to the assessment of research performance. Specifically, these authors combined traditional quantitative variables with analysis of the pioneering authors in a scientific area or research topic, the consistency of research over time, the number of papers with first authorship, and Scival topic prominence (including top quartiles).

In traditional bibliometrics, when authors use citation analysis to assess the impact of an article (e.g., when analysing an author’s or a university’s performance), the main indicator applied is the average number of citations per publication. In topic prominence analysis, the indicator applied is percentile-based [26]; that is, the higher the percentile, the greater the impact of the article [27]. In this context, the percentile reflects “the proportion of frequently cited publications, for instance, the proportion of publications that belong to the top 10% most frequently cited of their field” [28] (p. 372). Scival topic prominence is used to make predictions regarding a field of research. This new research indicator emerges from technology-related areas, and has been employed in the analysis of social science and business studies (i.e., tourism and hospitality) by Cardoso et al. [8,9,10]. By identifying research topics and capturing emerging subjects/topics in a specific area, topic prominence acts as a new performance indicator. It is useful in identifying whether a research topic is growing or declining, predicting whether it will grow or decline in the future, and indicating emerging topics of research [26]. In this context, according to Elsevier Publisher [29], topic prominence is considered to be a pointer that explains the prevailing momentum of a topic.

Moreover, as it indicates the impact of a paper, article or journal, topic prominence is a useful tool for researchers seeking funding. As argued by Wang and Shapira [30], high-impact articles (i.e., articles positioned in 90th and 95th percentiles) are far more likely to be associated with acknowledged funding when compared with low-impact articles. Therefore, topic prominence is normally used to support research funding [26]. Moreover, institutions seek to position themselves and gain notoriety through the impact of their publications. Therefore, topic prominence is a promising metric and has been increasingly employed for mapping scientific performance [31]. Given the potential of topic prominence, it was deemed an adequate tool for the present investigation.

## 3. Materials and Methods

The present investigation aimed to assess the global performance of neuromarketing studies. This objective was achieved through a set of methodological procedures, including a systematic search on neuromarketing and a combination of bibliometric analysis techniques. The following sub-sections address each of these procedures in detail.

### 3.1. Indicators and Methods Used

The methodological procedures employed in the present study included a combination of qualitative and quantitative analysis methods employed in different phases of the investigation, as in in previous bibliographic analyses by Cardoso et al. [8,9,10] and Ohniwa et al. [31]. The decision to use this approach was based on the premise that quantitative methods are more appropriate for drawing statistical inferences and comparisons, while qualitative methods are more suitable for discovering and generating theories [25]. In this context, several indicators, based on the contributions of previous studies [8,10] were employed in order to assess the performance of neuromarketing studies.

**Figure 1:**
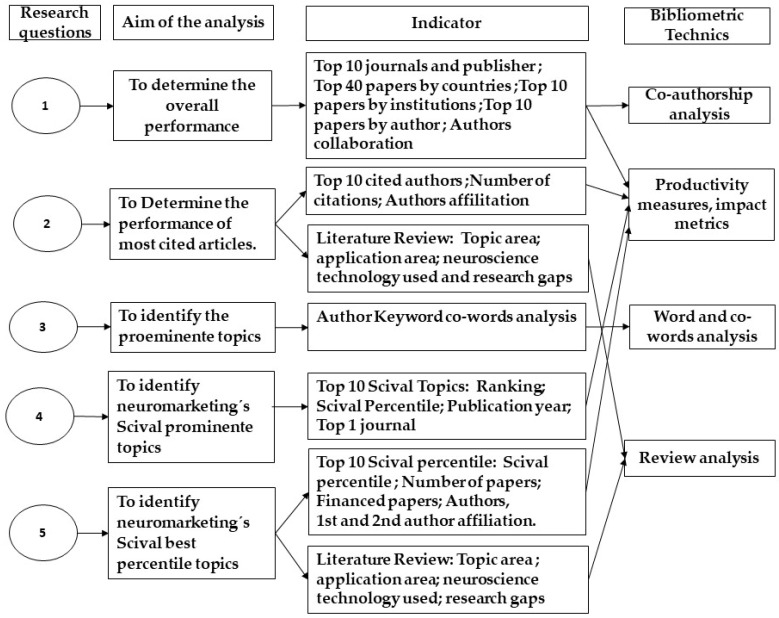
Summary of research questions, indicators, and technics used.

One such indicator was Scival topic prominence. As it had not previously been employed in bibliographic studies on the domain of neuromarketing, its application in the present investigation relied on contributions from studies in other research areas such as Klavans and Boyack [26] and Small et al. [32]. Other indicators that must be clarified include word growth and worth network; the use of such indicators was based on the work of Small et al. [32]. The methodological procedures adopted and the specific research questions that these indicators were intended to tackle are summarized in Figure 1.

### 3.2. Data Collection and Organization Procedures

The data collection procedures were carried out on 7 January 2021. The first step consisted of conducting a document search on Scopus for articles containing the exact key word (Limit to: exactkeywork) “neuromarketing” in the title, abstract, or keywords. The Scopus database is one of the largest high-quality abstract and citation datasets of peer-reviewed literature on the web, and is the only database that features Scival topic prominence [8,9,33]. Therefore, it was deemed an adequate source for the purposes of the present investigation.

A total of 318 articles were retrieved. The database was downloaded in Bibtex format from Scopus and subsequently screened with the help of R Studio software (version 1.2.5042) in order to eliminate duplicates and create a unified file. The database was later exported to R Bibliometrix 3.0., which was used for the network analysis in the same manner as previous studies [22,34]. The next step consisted of homogenizing the data and creating an Excel file to which SciVal topic prominence information was manually added, as Scopus does not include such data in the initial downloadable output. The Excel file was uploaded to DB Gnosis software, in which the content analysis was carried out. As argued by Cardoso et al. [8,10] when applied to bibliometric studies, content analysis allows for a clear view of the evolution of the literature on a given topic through the mapping of its scientific production. Therefore, it was deemed a useful technique in the context of the present investigation. Table 1 summarizes the main information obtained on the Scopus database.

### 3.3. Data Analysis Techniques and Procedures

Overall performance, analogous to the studies of Cardoso et al. [8,10], was accessed through rankings obtained via frequency counting using DB Gnosis software. The only exception to this was collaboration, which was assessed through network analysis carried out in R Bibliometrix, as proposed by Lima Santos et al. [25]. The performance of the most cited authors was assessed through DB Gnosis using citation frequency as an indicator. This body of literature was interpreted through categorical content analysis, which served to identify and describe the following variables: topic area, application area, neuroscience technology used, and research gaps addressed. The variables of analysis in the literature review analysis followed the results of Alsharif et al. [12,33]. Additionally, the affiliation of each author was checked during content analysis.

Following the methodology previously applied by Lima Santos et al. [25] and Cardoso et al. [10], performance of the prominent topics was accessed using two analyses in R Bibliometrix. The authors’ network and authors’ keyword network structures were performed in R Bibliometrix through Biblioshiny, which provides a web interface for R Bibliometrix. The authors’ network parameters used included normalization by inclusion. The cluster algorithm applied was betweenness. The analysis included 22 edges (excluding isolated nodes), and the output figure considered a minimum of two edges. The authors’ keyword network parameter used was normalization by association. Again, the applied cluster algorithm was betweenness, the analysis included 22 edges (excluding isolated nodes), and the output figure considered a minimum of two edges. Finally, Scival topic prominence and the prominence percentile were accessed using the methodology proposed by Cardoso et al. [8,9,10], that is, frequency rankings carried out using DB Gnosis.

## 4. Results

### 4.1. Neuromarketing: Overall Research Performance

The retrieved articles are published in 212 different journals, with a high level of dispersion between them. As shown in Table 2, two journals, “Frontiers in Neuroscience” and “Frontiers in Psychology”, stand out with nine articles each. Accordingly, four publishers stand out for having ten or more papers in neuromarketing field.

In terms of publications by country, as detailed in Table 3 Spain leads the ranking of the most productive countries with 48% of neuromarketing publications, followed by the United States and China. Spanish researchers focus on advertising, particularly online advertising and TV Commercials, as well as on the role of image and sound effects on viewer behavior. The articles published by Spanish research centers use eye tracking, galvanic skin response, electroencephalography (EEG) and functional magnetic resonance imaging (fMRI) technology. The United States, a close second, is responsible for 39% of all Scopus publications on neuromarketing. American research centers focus on studying TV Commercials. With a considerable gap from the top two countries, China ranks third with 24% of all Scopus publications on neuromarketing. The most-researched topics in Chinese institutions are consumer association and branding strategy, both of which are linked to TV commercials, color association, and tourism.

Regarding the most productive Institutions in neuromarketing research, the results are summarized in Table 4. Complutense Madrid University (Spain) leads the ranking with 21 papers, followed by Roma la Sapienza University (Italy) and Zhejiang University (China) with 19 articles each. As expected, and considering the results shown in Table 3, in addition to the leading institution Spain has three other universities in the top ten: Granada University is ranked sixth with twelve papers, while Valencia Polytechnic University and Vigo University share the seventh position with two other institutions (eight papers each).

Regarding individual authors’ performance (using articles fractionalized/co-authorship and first authorship papers as indicators), as displayed in Table 5, two authors share the first position in terms of total articles published. The first, Babiloni F., is Professor of Physiology and Director of the Industrial Neuroscience lab in Rome’s Sapienza University, Italy. He has an average fractional article participation items of 1.20 and is a co-author of nine neuromarketing papers (although not being the first author on any), which mostly address TV Commercials. The other author sharing the first position, Ma Q., is a Professor in the School of Management at Zhejiang University, China and a member of the Neuromanagement Lab. He is the first author on six papers, and a co-author of another three. His research encompasses the topics of neuromanagement, branding (consumer behavior), purchase intention, price perception, and emotion.

The third most prominent author is Vecchiato G., from Rome University. He is the first author of seven papers and researches TV Commercials, focusing on emotion and attention. The fourth most prominent author is Crespo-Pereira V., from the University of Ecuador. The first author of two papers (and co-author of another seven), his research is about the entertainment industry, mainly TV entertainment and the emotions it arouses in the audience. Regarding first authorships, Vecchiato G. stands out, as he is the first author on seven of the eight papers he published.

**Figure 2:**
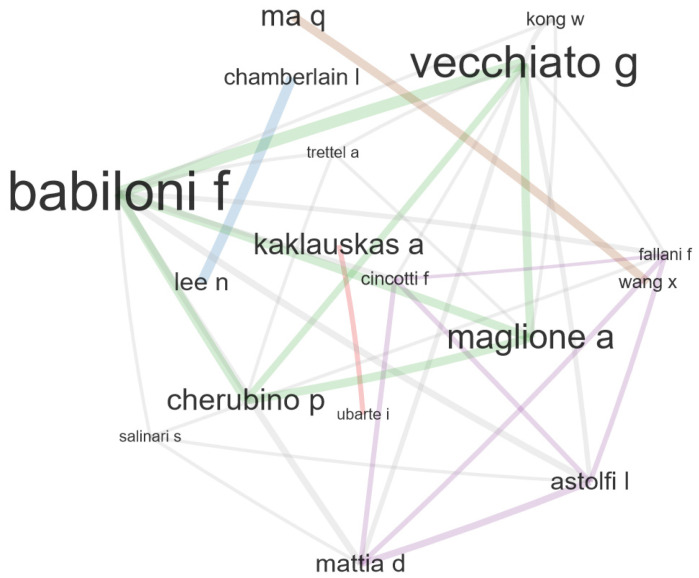
Authors’ collaboration.

The analysis of the worldwide neuromarketing authors’ collaboration network, which is graphically represented in Figure 2, reveals two strong connections between two groups of four authors. The first group is centered on Babiloni F. and Vecchiato G., both from Rome’s Sapienza University. This group shows a higher degree of connectivity and more consistent and direct connection. Vecchiato G., who is first author on most of the articles, acts as a broker by linking different clusters of Italian authors within the network. Amongst the authors brokered by Vecciato G., two stand out: Maglione A., from the Department of Economics and Marketing of the “IULM” University, and Cherubino P., from BrainSigns, Italy. Both are connected to Babiloni F. as well as with each other. This research group applies neuroelectrical, cognitive, and emotional variables to TV commercials.

The second collaboration group stems from Astolfi L., of the Department of Computer Science at Rome’s Sapienza University. This author connects to Fallani F. from the Paris Brain Institute (ICM), France; Mattia D. from the Department of Neuroscience at the University Tor Vergata, Italy; and Cincotti F. from the Center di Ricerca de La Sapienza Per l’Analisi dei Modelli e dell’Informazione, Italy. This research group studies brain responses to TV commercials. Analogous to the first group, it consists exclusively of Italian researchers.

### 4.2. Performance of the Most Cited Neuromarketing Articles

Neuromarketing studies aim to better understand consumer preferences by gaining access to potentially unconscious neural and physiological responses [35]. They aim to predict or even manipulate consumer behavior and decision making [36]. Neuromarketing has been used for “neuro forecasting” [36], predicting marketplace responses [37,38], fine tuning segmentation [36], and nudging consumer behaviors [36]. Table 6 details the Top ten neuromarketing papers in terms of citations, and Table 7 presents the topic area, application area, neuroscience technology used, and research gap addressed by each of those papers.

Among the top ten articles, three [39,40,41] consist of introductions to neuromarketing. The majority of articles focus on predicting consumer behavior and preferences in various industries (i.e., music, movies, snack foods) [11,37,38,42,43,44]. Another frequently-addressed topic is the generalizability of individual fMRI results to market sales data [31,37]. In terms of neuroscience techniques, these researchers rely on fMRI, EEG, skin conductance and eye tracking to collect data on participant responses to stimuli. The only exception in this regard is Lopes et al. [1], which was included due to the use of the keyword neural networks.

### 4.3. Performance of Most Prominent Neuromarketing Topics: Author Word Network

**Figure 3:**
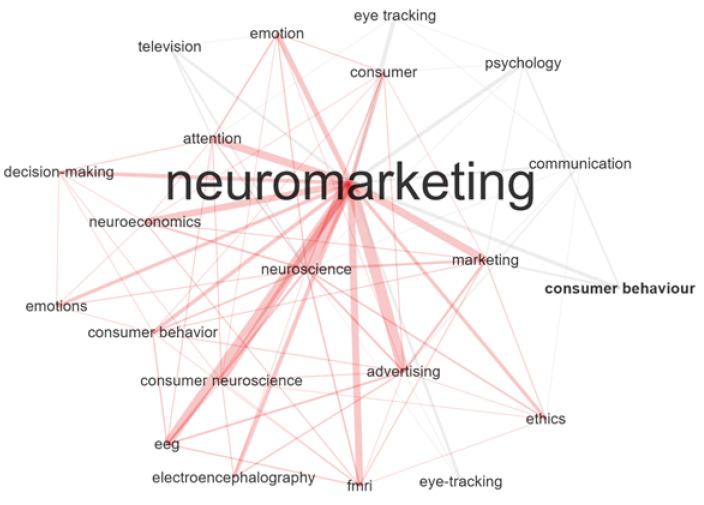
Author keywords: co-word analysis.

To explore the most prominent themes in neuromarketing research from 2007 to 2020, a word network structure was performed considering 22 nodes with a minimum of two edges, as represented in Figure 3. “Neuroscience”, with a betweenness score of 6.14 of, maintains a network of interconnections with most of the themes in the network. Accordingly, “Advertising” is the topic with the highest proximity (2.32 betweenness). This analysis highlights the interconnections between “advertising”, “marketing”, “neuroscience”, “consumer neuroscience”, “EEG”, and “electroencephalography”, which define this thematic network.

Another strong thematic network (1.26 betweenness) is drawn through the theme of “attention”, with which several keywords are interconnected, including “emotion”, “decision-making”, “neuroeconomics”, “emotions”, “EEG”, and “electroencephalography”. Moreover, “emotions” is directly linked with “neuroeconomics” and “decision-making”, while “emotion” (with 0.2 betweenness) is directly linked with “attention” and “consumer”. These connections, however, are not strong. This low connectivity index is typical of emerging topics.

Other topics with low betweenness indexes (0), and therefore weak connectivity links, are “television”, “consumer behavior”, and “eye tracking”. Naturally, in the context of neuromarketing these are emerging topics. Regarding the nodes related to the technologies used in neuromarketing research, “EEG”, “FMRI”, and “eye tracking” relate to “consumer” and “advertising”, which reflects the use that is normally made of such techniques.

### 4.4. Performance of Prominent Scival Neuromarketing Topics 

SciVal topic prominence performance is operationalized through the outputs of several variables, which are summarized in Table 7. The most relevant result is that 55% of all scientific production on neuromarketing is clustered under the “Neuromarketing | Neurosciences | TV Commercial” Scival topic prominence, which has a 94.432 prominence percentile. This means that it is in the top 5% of all topics in the world, with high levels of CiteScore, citations, and views. The topic emerged in 2007, after which publications have consistently increased up to 2020. An analysis of this topic on the Scival platform (performed in 13 March 2021) reveals that 640 articles were published worldwide from 2010 to 2020, 27% of which were published in neuromarketing field. This is a high figure compared to overall world production. The leading country in this research topic is Spain, through Complutense University.

The other topics that follow the ranking of neuromarketing research mostly emerged after 2010. Most of these emerging topics have high prominence percentiles, which indicates that they generate high levels of research interest. For instance, the topic “Subjective Well-being | Happiness | Life Satisfaction” emerged in 2020, and has a 98.770 prominence percentile.

### 4.5. Neuromarketing

The 99th–100th percentile, that is, the 1% best percentile on the worldwide momentum and visibility of topics, includes 17 studies. Information on these topics, namely, the authors who publish on each topic, whether they got funding for their research, and first and second author affiliations, is summarized on Table 8. Accordingly, information on the individual studies, namely, the topic area, application area, neuroscience technology used, and research objective/gap, are summarized on Table 9.

The articles within this percentile can be classified into three different categories: audience response studies, methodology improvement studies, and literature reviews. Audience response studies [45,46,47,48,49,50,51,52] explore the impact of marketing campaign pieces on the consumer decision-making process and examine consumer responses and preferences. These studies are applied to many consumer industries and specific markets, such as the coffee industry and hotel industry, and are applied to specific marketing functions, such as corporate communication and brand image.

Methodology improvement studies [1,53,54,55,56,57,58] propose methods for enhancing the accuracy of object recognition and classification by combining neuromarketing techniques with traditional marketing research methods. These studies are applied to specific goals of neuromarketing methods, such as emotional face retrieval and facial expression recognition. Others focus on certain consumer decision scenarios and situations, such as grocery shopping in supermarkets. Others focus on the media through which information is conveyed, such as audio, video, or still images. Finally, literature review articles [59,60] explore EEG applications in research, consumer privacy concerns, and the regulatory environment.

## 5. Conclusions

The present study aimed to examine the global performance of neuromarketing studies. To address this general goal, five specific objectives were adopted:

(1) To determine the overall performance of neuromarketing research;
(2) To determine the performance of the most cited articles;
(3) To identify the most prominent topics within the neuromarketing field;
(4) To identify the prominent Scival topics on neuromarketing; and
(5) To identify the best Scival topics on neuromarketing by percentile.

To achieve these objectives, scientific production on neuromarketing was mapped through a mixed-method bibliographic approach.

Regarding the first objective, 318 papers containing the word “neuromarketing” were published in the Scopus database between 2010 and 2020. In terms of leading journals, the ranking is led by Frontiers in Neuroscience and Frontiers in Psychology, closely followed by Profesional de la Informacion. In terms of publications by country, Spain leads the ranking, followed by the United States, China, and Italy. These results are reflected in those on the leading universities, as the Complutense Madrid University leads the ranking with 21 articles, followed by Rome’s la Sapienza University, Italy and Zhejiang University, China.

Although Italy is only fourth in the country ranking, Italian researchers stand out in terms of individual productivity as well as collaboration. The first, third and fourth most productive researchers are Italian and work in Italian research centers. Accordingly, the global collaboration network on neuromarketing is dominated by two very prominent groups of Italian researchers.

Regarding the second objective, the number of introductory articles within the top ten papers in terms of citation demands attention, as it indicates that neuromarketing remains a developing topic. This is somewhat expected, given how recently the subject has come to prominence. Another popular research goal is predicting consumer behavior and preferences in different scenarios, which is line with the general goals of neuromarketing studies, that is, to better understand consumer preferences by gaining access to potentially unconscious neural and physiological responses [36]. In neuromarketing studies, this goal is pursued via the application of neuroscience technologies. The present study’s findings point to fMRI, EEG, skin conductance, and eye tracking as the most employed neuroscience technologies in neuromarketing studies.

Regarding the third specific objective, most prominent topics within the subject are connected to “Neuroscience” and have some degree of approximation with “Advertising”. The network of prominent topics provides an overview of the main neuroscience techniques and technologies used in these studies, namely, EEG/electroencephalography. Once again, this reflects the very nature of neuromarketing studies, which apply neuroscience techniques to understand and predict consumer behavior in response to certain stimuli. Naturally, a popular source of such stimuli (as these results corroborate) is advertising, especially television advertising, as the topic is highlighted in the network. Therefore, these studies have the potential to unveil the effectiveness of advertising techniques and generate good practices on how best to generate the expected response from consumers in specific industries.

The findings stemming from the fourth objective largely corroborate those of the third, as they show that over half of the scientific production on neuromarketing is clustered under the “Neuromarketing | Neurosciences | TV Commercial” Scival topic prominence, which is within the top 5% of all topics in the world in terms of views and citations. Therefore, this cluster, and consequently neuromarketing research in general, arouses high levels of research interest and has significant potential for funding.

Finally, the findings from objective five provide a list of the very best rated topics in terms of visibility and momentum. Articles within the top 1% percentile include audience response studies, methodology improvement studies, and literature reviews. Audience response studies make up the bulk of neuromarketing research. They employ the technologies highlighted in the network analysis to analyse and predict consumer reactions, especially to advertising, as well as to real-world consumption situations such as hotel stays and grocery shopping.

Methodology improvement studies attempt to refine the methods applied in the previous category, providing best practices on how to analyse consumer reactions and behaviour more precisely and reliably. Finally, literature review studies, as in any other area, summarise and systematise the state of the art on the topic. The highlighted role they play in the neuromarketing field is (analogous to introductory studies) indicative of the fact that the topic is developing quickly, which calls for constant updating of systematic reviews.

### Contributions, Implications, and Future Research

The present investigation consisted of a comprehensive bibliometric analysis of academic production on neuromarketing. To this end, in addition to traditional bibliometric indicators, SciVal Topic prominence was examined, which had not been carried out in previous neuromarketing reviews. Therefore, this study provides a more detailed understanding of the state of the art in this area as well as of trending and emerging topics and those with the highest potential for publication. For neuromarketing researchers, the present study reveals gaps in research as well as emerging subjects by identifying research topics that are growing, making it possible to identify future lines of research. This should be quite useful for researchers and research centers aiming to conduct investigatory projects in this area. Moreover, previous studies have shown that research impact and topic prominence are positively related to the proportion of financed research projects within a topic. Therefore, the findings of the present study are particularly useful for researchers seeking financing for such projects.

The present research provides a contribution for future bibliometric studies. It shows that the method proposed by Cardoso et. al. [10] to map a country’s research performance in a certain area of research—in that case, Switzerland, and Tourism and Hospitality—can be employed to map the worldwide scientific production in each area. Therefore, future studies are encouraged to employ this method, and particularly the novel measure used here (i.e., SciVal Topic prominence analysis), in order to assess research production in other areas and to update the state of the art on neuromarketing in the future.

Regarding future bibliographic studies on neuromarketing, the present research raises some questions that could be addressed by future works. In terms of worldwide collaboration, for instance, the present research highlights the clear domination of Italian research and research centres, although Italy is only fourth in the ranking of most productive countries. However, it was beyond the scope of this study to explore the reasons behind this phenomenon. In this context, future studies could further investigate these reasons, and determine whether research links aim to combine the expertise of researchers in each centre, the technologies owned by each institution, or whether they merely reflect the contact networks and academic histories of the involved researchers.

## Figures and Tables

**Table 1:** Description of Scopus neuromarketing data.

MAIN INFORMATION ABOUT DATA	Results
Timespan	2007:2020
Sources (Scopus Journals)	212
Documents	318
Average years from publication	3.86
Average citations per document	14.78
Average citations per year per document	2313
References	15.246
DOCUMENT TYPE	
Article	318
DOCUMENT CONTENTS	
Keywords Plus (ID)	989
Author’s Keywords (DE)	913
AUTHORS	
Authors	850
Author Appearances	1072
Authors of single-authored documents	50
Authors of multi-authored documents	800
AUTHOR COLLABORATION	
Single-authored documents	52
Documents per Author	0.374
Authors per Document	2.67
Co-authors per Document	3.37
Collaboration Index	3.01
SCIVAL TOPIC PROMINENCE	315

**Table 2:** Top ten journals and publishers in neuromarketing research productivity.

Rank	Journal	Articles	Rank	Publisher	Articles
1	Frontiers in Neuroscience	9	1	Emerald Group Publishing LTD.	20
2	Frontiers in Psychology	9	2	Frontiers Media S.A.	15
3	Profesional de la Informacion	7	3	Elsevier INC.	10
4	Comunicar	6	4	MDPI AG	10
5	Journal of Neuroscience Psychology and Economics	6	5	Elsevier LTD	9
6	Cogent Psychology	5	6	Routledge	7
7	Journal of Business Research	5	7	Cogent OA	5
8	Revista Latina de Comunicacion Social	5	8	Sage Publications INC.	5
9	Asia Pacific Journal of Marketing and Logistics	4	9	Springer New York LLC	5
10	Behavioral Sciences	4	10	American Psychological Association INC.	4

**Table 3:** Top twenty countries in neuromarketing research productivity.

Country	Absolute Frequency	Country	Absolute Frequency
Spain	154	Canada	14
USA	124	Ecuador	13
China	75	Slovakia	11
Italy	57	Colombia	10
Germany	54	Mexico	10
UK	52	Ukraine	10
South Korea	43	Austria	9
Turkey	38	Portugal	8
Australia	33	Saudi arabia	7
Brazil	33	Belgium	6
Lithuania	28	Czech Republic	6
Netherlands	26	Chile	5
Iran	25	Singapore	5
Japan	25	Switzerland	5
Malaysia	23	Vietnam	5
Romania	21	Bangladesh	4
Denmark	20	Cyprus	4
France	19	Finland	4
India	16	New Zealand	4
Poland	15	Peru	3

**Table 4:** Top ten institutions in neuromarketing research productivity.

Authors Institutions Affiliations	Articles
Complutense Madrid University, Spain	21
Rome la Sapienza University, Italy	19
Zhejiang University, China	19
Sungkyunkwan University, South Korea	15
Vilnius Gediminas Tech University, Lithuania	14
Ningbo University, China	13
Islamic Azad University, Iran	12
Granada University, Spain	12
Swinburne Polytechnic University, Australia	8
Oxford University, the UK	8
Valencia Polytechnic University, Spain	8
Vigo University, Spain	8

**Table 5:** Top ten authors’ neuromarketing research productivity.

Authors	Articles	Articles Fractionalized	First Authorship Papers	Affiliation
Babiloni F.	9	1.20	No	Sapienza University of Rome, Italy
Ma Q.	9	2.45	6	Zhejiang University, China
Vecchiato G.	8	1.22	7	Sapienza University of Rome, Italy
Crespo-Pereira V.	7	3.33	4	Pontificia Universidad Católica del Ecuador, Ecuador
Lee N.	7	2.19	2	University of Warwick, Coventry, United Kingdom
Chamberlain L.	6	1.69	No	Aston University, United Kingdom
Grigaliunaite V.	6	3.00	2	Vytautas Magnus University, Lithuania
Kaklauskas A.	6	1.07	5	Vilnius Gediminas Technical University, Lithuania
Maglione A.	6	0,90	No	Department Economics and Marketing, “IULM” University
Pileliene L.	6	3.00	2	Vytauto Didziojo Universitetas, Lithuania
Ramsoy T.	6	2.45	No	Neurons Inc, Copenhagen, Denmark
Wang X.	6	1.53	No	Zhejiang University, China
Cherubino P.	5	0.65	No	BrainSigns, Italy
Kong W.	5	0.98	1	Hangzhou Dianzi University, China

**Table 6:** Top ten authors in neuromarketing paper citations.

Authors	Number of Citations	Affiliations	Topic Area	Application Area	Neuroscience Technology Used	Research Aim or Research Gap
Lee et al. (2007)	307	Aston Business School, Aston University, UK	Introduction and review	not applicable	not applicable	To provide a scholarly perspective on neuromarketing
Lopes et al. (2017)	290	Universidade Federal do Espírito Santo, Brazil	Methodology improvement	Fascial expression recognition	not applicable	To propose a new method for improving the accuracy of facial expression recognition
Khushaba et al. (2013)	195	University of Technology, Sydney (UTS), Australia	prediction of consumer behavior	Crackers	EEG and eye tracking	To analyze EEG spectral changes in a choice context to measure specific features of the choice options
Reimann et al. (2010)	194	University of Southern California, USA	Consumer choice preference	Packaging	fMRI	The neural underpinnings of aesthetic packaging experiences is nonexistent in the literature
Plassmann et al. (2012)	172	INSEAD, France	Literature review	not applicable	not applicable	How neuroscience can advance consumer psychology concerning brands
Dimoka et al. (2011)	149	Temple University, Philadelphia, USA	Research commentary	not applicable	not applicable	To introduce cognitive neuroscience theories, methods, and tools to IS researchers
Falk et al. (2012)	144	University of Michigan, USA	Consumer responses	TV campaigns	fMRI	Can the neural responses of individuals predict the behavior of a population?
Berns & Moore (2010)	121	Emory University, Atlanta, USA	Prediction of consumer behavior	The music industry	fMRI	Can the neural responses of individuals predict subsequent market results?
Boksem and Smidts (2015)	106	Rotterdam School of Management, Netherland	Consumer choice preference	Movie traiers	EEG	To investigate whether neural measures contribute to the prediction of commercial success beyond stated preference measures
Ohme et al. (2009)	104	Polish Academy of Sciences, Poland	Consumer responses	TV Ads	EEG, EMG, and skin conductance	To investigate whether neurophysiological measures can capture differences in consumer reactions

EEG—Electroencephalogram; EMG—Electromyography; fMRI—Functional magnetic resonance imaging.

**Table 7:** Top ten: Scival Topic Prominence.

R	Scival Topic Prominence:	AF	RF	SP	Publication Year	TOP 1 Journal
1	Neuromarketing | Neurosciences | TV Commercial	175	0.55	94.432	2020-19; 2019-37; 2018-15; 2017-28; 2016-10; 2015-13; 2014-10; 2013-8; 2012-13; 2011-6; 2010-6; 2009-3; 2008-4; 2007-3	Frontiers in Neuroscience-5Frontiers In Psychology-5
2	Celebrity Endorsement | VIP | Purchase Intention	6	0.019	95.662	2020-2; 2017-2; 2016-1; 2010-1	Scientific Annals of Economics and Business-2
3	Emotion Recognition | Electroencephalography | Brain Computer Interface	6	0.019	98.281	2018-2; 2016-3; 2014-1	Acta Universitatis Agriculturae et Silviculturae Mendelianae Brunensis-1
4	Near-infrared Spectroscopy | Diffuse Optical Tomography | brain Computer Interface	6	0.019	98.186	2020-1; 2019-1; 2018-2; 2017-1; 2016-1	Advances In Experimental Medicine and Biology-1
5	Motor Imagery | Brain Computer Interface | Visual Evoked Potentials	5	0.015	99.769	2019-2; 2017-1; 2016;1; 2015-1	Journal Of Advanced Computational Intelligence and Intelligent Informatics-2
6	Implicit Association Test | Implicit Measures | Avoidance Conditioning	3	0.009	98.521	2020-1; 2016-1; 2013-1	Behavioral Sciences, MDPI-1
7	Message Sensation Value | Sensation Seeking | Public Service Announcements	3	0.009	78.288	2017-1; 2013-2	Historia Y Comunicacion Social-1
8	Sentence Comprehension | Left Anterior Negativity | Syntactic Processing	3	0.009	96.494	2019-1; 2016-1; 2012-1	Neuroscience Letters-2
9	Sport Sponsorship | Ambush Marketing | Sponsor	3	0.009	93.018	2020-1; 2019-1; 2018-1	International Journal of Sports Marketing and Sponsorship-2
10	Subjective Well-being | Happiness | Life Satisfaction	3	0.009	98.770	2020-1; 2019-2	Energies, MDPI-1

Ranking (R); Absolute Frequency (AF); Relative Frequency (RF); Scival Percentile (SP).

**Table 8:** Top ten Scival topics by best percentile: funding and author affiliations.

R	SP	NP	Topic	Authors	F	1st and 2nd Author Affiliation
1	99.922	2	Cause-related Marketing | corporate Social Performance | Corporate Philanthropy	Lee (2016); Mañas-Viniegra et al. (2020)	Yes-2	Sungkyunkwan University—South Korea; Complutense University of Madrid—Spain
2	99.941	1	Electronic Word-of-mouth | Online Reviews | Brand Community	Hsu and Cheng (2018)	No	Minghsin University of Science and Technology—Taiwan
3	99.858	1	Product-service Systems | service Economy | Value Co-creation	Zhao et al. (2019)	Yes	Heilongjiang University—China Beihang University—China
4	99.769	5	Motor Imagery | brain Computer Interface | Visual Evoked Potentials	Wójcik et al. (2015) Fan and Touyama (2016) Fujita and Touyama (2017) Libert and Van Hulle (2019) Chi Qin et al. (2019)	Yes-1 No-4	Maria Curie-Skłodowska University—Poland Toyama Prefectural University—Japan KU Leuven-University of Leuven—Belgium Universiti Sains Malaysia—Malaysia
5	99.718	1	Human-robot Interaction | Humanoid Robot | uncanny	Chung et al. (2020)	Yes	Yonsei University Health System, South Korea Seoul National University, South Korea
6	99.568	2	Prefrontal Cortex | prediction Error | Reward	Heinonen and Briesemeister. (2018). Çakir et al. (2018)	No	Laurea University of Applied Sciences, Espoo, Finland Middle East Technical University, Ankara, Turkey MEF University, Istanbul, Turkey
7	99.470	1	Privacy Concerns | online Shopping | Social Commerce	Rapp et al. (2009)	Yes	University of Nebraska, USA Villanova School of Business, USA
8	99.393	1	Emotion Recognition | Facial Expression | Smile	Lopes et al. (2017)	Yes	
9	99.166	2	Servicescape | Customer Experience | Mall	Rodas-Areiza and Montoya-Restrepo (2018) Zavadskas et al. (2019)	Yes-2	Instituto Tecnológico Metropolitano, Medellín, Colombia Universidad Nacional de Colombia, Medellín, Colombia Vilnius Gediminas Technical University, Vilnius, Lithuania
10	99.150	1	Journalism | News Production | Journalistic Practices	Mañas-Viniegra et al. (2020)	Yes	Complutense University of Madrid, Spain

Ranking (R); Scival Percentile (SP); Number of papers (NP); Financing (F).

**Table 9:** Articles within the top ten Scival topics by best percentile: topic area, application area, neuroscience technology, and research objectives.

Authors	Topic Area	Application Area	Neuroscience Technology Used	Research Aim or Research Gap
Lee (2016)	Audience response	Coffee	EEG	The emotional mechanism of empathy and the neural correlates underlying the positive consumer reactions to pro-social marketing
Mañas-Viniegra et al. (2020)	Audience response	Corporate communication and brand image	Eye tracking and galvanic skin response	Do audiences have different attention and emotions toward corporate purpose message and corporate visual identity?
Hsu and Cheng (2018)	Audience response	Hotels	EEG	To compare brainwave results when viewing videos with and without subliminal stimuli
Zhao et al. (2019)	Audience response	Camera	EEG	To understand the roles of elements and design in meeting customer demand regarding product and service systems
Wójcik et al. (2015)	Methodology improvement	Equipment failure	Not applicable	To recommend a solution for fixing an Emotive EEG technical problem
Fan and Touyama (2016)	Methodology improvement	Emotional face retrieval	EEG	To improve the accuracy of emotional face retrieval classification
Fujita and Touyama (2017)	Methodology improvement	Audio	EEG, eye tracking (EOG signals)	To develop a method of single-shot multimedia content evaluation based on collaborative P300 signals in order to improve accuracy
Libert and Van Hulle (2019)	Methodology improvement	Video	EEG	To develop a method to predict video behavior and viewing interest
Chi Qin et al. (2019)	Literature review		EEG	To explore and categorize EGG applications in research
Chung et al. (2020)	Audience responses	Recommendation agents	EEG	Do people prefer natural virtual agents (human) or artificial intelligences?
Heinonen and Briesemeister (2018)	Methodology improvement	Research method	fMRI	To shorten fMRI time using conjoint analysis
Çakir et al. (2018)	Methodology improvement	Supermarket products	fNIRS	To develop a neurophysiologically-informed model of purchasing behavior based on fNIRS measurements
Rapp et al. (2009)	Commentary	Consumer privacy concerns	Not applicable	To review consumer privacy concerns and regulatory environment, and to make recommendations
Lopes et al. (2017)	Methodology improvement	Facial expression recognition	Not applicable	To propose a new method to improve the accurancy of facial expression recognition
Rodas-Areiza and Montoya-Restrepo (2018)	Methodology improvement	Fascial cream	Face reader, EEG, and eye tracking	To propose a framework to incorporate and measure the impact of sensory marketing
Zavadskas et al. (2019)	Methodology improvement	A fair	Facial emotion tracker, temperature analysis device, a respiration sensor	The integration of the emotional and physiological states of potential buyers, their valence and arousal, and their affective attitudes into the analysis of the one-to-one marketing process.
Mañas-Viniegra et al. (2020)	Audience response	Journalism	Eye tracking and galvanic skin response	The existence of differences between a drone recording and a conventional news recording and the cause of the difference

EEG: Electroencephalogram; EMG: Electromyography; EOG: Electrooculography; fMRI: Functional magnetic resonance imaging; fNIRS: Functional near-infrared spectroscopy.
